# Induction of D-xylose uptake and expression of NAD(P)H-linked xylose reductase and NADP + -linked xylitol dehydrogenase in the oleaginous microalga *Chlorella sorokiniana*

**DOI:** 10.1186/s13068-014-0125-7

**Published:** 2014-10-03

**Authors:** Yubin Zheng, Xiaochen Yu, Tingting Li, Xiaochao Xiong, Shulin Chen

**Affiliations:** LJ Smith 258, Biological Systems Engineering, Washington State University, Pullman, WA 99164 USA

**Keywords:** *Chlorella sorokiniana*, Microalgae, D-xylose, Xylose reductase, Xylitol dehydrogenase

## Abstract

**Background:**

The heterotrophic and mixotrophic culture of oleaginous microalgae is a promising process to produce biofuel feedstock due to the advantage of fast growth. Various organic carbons have been explored for this application. However, despite being one of the most abundant and economical sugar resources in nature, D-xylose has never been demonstrated as a carbon source for wild-type microalgae. The purpose of the present work was to identify the feasibility of D-xylose utilization by the oleaginous microalga *Chlorella sorokiniana*.

**Results:**

The sugar uptake kinetic analysis was performed with ^14^C-labeled sugars and the data showed that the D-glucose induced algal cells (the alga was heterotrophically grown on D-glucose and then harvested as D-glucose induced cells) exhibited a remarkably increased D-xylose uptake rate. The maximum D-xylose transport rate was 3.8 nmol min^−1^ mg^−1^ dry cell weight (DCW) with *K*_*m*_ value of 6.8 mM. D-xylose uptake was suppressed in the presence of D-glucose, D-galactose and D-fructose but not L-arabinose and D-ribose. The uptake of D-xylose activated the related metabolic pathway, and the activities of a NAD(P)H-linked xylose reductase (XR) and a unique NADP^+^-linked xylitol dehydrogenase (XDH) were detected in *C. sorokiniana*. Compared with the culture in the dark, the consumption of D-xylose increased 2 fold under light but decreased to the same level with addition of 3-(3,4-dichlorophenyl)-1,1-dimethylurea (DCMU), indicating that extra chemical energy from the light-dependent reaction contributed the catabolism of D-xylose for *C. sorokiniana*.

**Conclusions:**

An inducible D-xylose transportation system and a related metabolic pathway were discovered for microalga for the first time. The transportation of D-xylose across the cell membrane of *C. sorokiniana* could be realized by an inducible hexose symporter. The uptake of D-xylose subsequently activated the expression of key catalytic enzymes that enabled D-xylose entering central metabolism. Results of this research are useful to better understand the D-xylose metabolic pathway in the microalga *C. sorokiniana* and provide a target for genetic engineering to improve D-xylose utilization for microalgal lipid production.

## Background

Microalgal biomass is considered as potential feedstock for making biofuels and high-value chemicals [1,2]. Phototrophic culture of microalgae suffers from inherent low density due to self-shading, resulting in a high harvesting cost [3]. Growing microalgae heterotrophically or mixotrophically by feeding with organic carbons shows great potential to overcome this limitation. High growth rate, cell density and biomass productivity could be achieved for some microalgal species [4,5]. Based on the analysis of energetics and carbon metabolism during microalgae growth, Yang *et al*. [6] demonstrated that heterotrophic and mixotrophic cultures produced more ATP and biomass from the energy supplied than the phototrophic culture. However, addition of organic carbon increases cost and makes the process economically unfeasible. Although some low value materials have been explored in order to reduce the cost, such as crude glycerol, acetate, food waste and cassava starch [4,7,8], the availability and sustainability of the organic carbon supplies remain limiting factors for large-scale applications.

Lignocellulosic materials are promising feedstocks due to their abundance and relatively low cost [9]. Mono-sugars can be obtained from hydrolysis of lignocellulosics and serve as carbon sources for the heterotrophic or mixotrophic culture of microalgae. D-glucose (mainly from cellulose) and D-xylose (from hemicellulose) are two major carbohydrates in the lignocellulosic hydrolysates. D-glucose has been proven as a suitable carbon source for the growth of many microalgae, such as *Crypthecodinium cohnii*, *Schizochytrium* sp., *Nitzschia laevis*, *Euglena gracilis*, *Galdieria sulphuraria* and *Chlorella* species [10]. Li *et al*. [11] successfully demonstrated that the microalga *Chlorella pyrenoidosa* could be cultured for lipid production on rice straw hydrolysates primarily containing D-glucose. The mechanism of D-glucose uptake in microalgae has also been determined by using *Chlorella* as the model strain. Tanner [12] revealed that uptake of D-glucose by *Chlorella* was based on an inducible hexose/H^+^ symport system. In the presence of the inducer D-glucose, *Chlorella* could synthesize the transporter in only 15 minutes and the uptake rate could increase more than 400-fold [13]. After being transported into the cells, the assimilated D-glucose is metabolized via the Embden-Meyerhof Pathway (EMP) and the Pentose Phosphate Pathway (PPP) [14].

Although culturing microalgae with D-glucose feeding has been intensively studied, the report on D-xylose utilization is very limited. Hawkins [15] screened a *Chlorella* strain with the capability to grow on D-xylose mixotrophically after ultraviolet (UV) irradiation. However, wild-type microalgae with the capability to utilize D-xylose as the sole carbon source have not been reported. A lack of efficient uptake system and/or the related metabolic pathway may be the major reasons for this. Neish [16] evaluated various carbohydrates on the growth of *Chlorella vulgaris*. The results showed that D-glucose, D-fructose, D-galactose and β-glucosides were good sources of carbon and energy for algal growth, but pentose sugars including D-xylose caused little or no simulation of growth. Similarly, Samejima and Myers [17] found that *C. pyrenoidosa* and *Chlorella ellipsoidea* could hardly grow on xylose although glucose and galactose supported continued growth. Moreover, Hassall [18] demonstrated that D-xylose could not be used as a substrate for chemosynthesis by the microalga *C. pyrenoidosa*, on the contrary, it acted as a specific inhibitor for the photosynthesis. In the presence of 0.5% D-xylose (33 mM), cell division was arrested and the color of microalgae was lost within a few days.

In our previous study, we reported a green microalga, *Chlorella sorokiniana* (UTEX 1602), with great potential for biofuel production due to its high growth rate, cell density and lipid content resulting from feeding with D-glucose [19,20]. However, the utilization of D-xylose, the second most abundant sugar in lignocellulosic biomass, was also critically required for cost-effective microalgal biofuel production. The aim of this study was to investigate D-xylose utilization by *C. sorokiniana* and the related metabolic pathway. Firstly, a strategy was devised for D-xylose utilization by the microalgae. Then, uptake kinetics was analyzed for the induced algal cells. The effects of different mono-sugars were examined on D-xylose uptake. Thereafter, enzyme assay was performed to detect the related metabolic enzymes. The enzyme characteristics including pH, temperature and cofactors were evaluated. Finally, the effect of light on D-xylose utilization was studied.

## Results

### D-xylose uptake in *C. sorokiniana*

**Figure 1:**
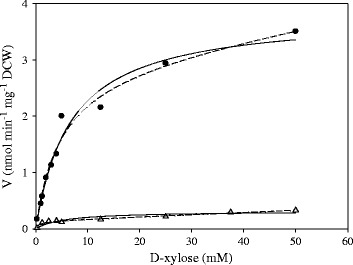
**D-xylose uptake kinetics of induced (black circle) and non-induced (white triangle)** ***C. sorokiniana*** **using two different models.** Model I (single-carrier, solid line) and Model II (two-carrier, dash line).

The consumption of D-xylose by *C. sorokiniana* was observed in the D-glucose induced algal cells (the alga was grown on D-glucose heterotrophically and then harvested as D-glucose induced cells). The kinetic parameters of two different models for D-xylose transport in *C. sorokiniana* are listed in Table 1. For induced algal cells, there was no significant difference of variances between these two models. As seen in Figure 1, the two-carrier model fitted the experimental data better than the single-carrier model. However, the *V*_*max2*_ and *K*_*m2*_ of the two-carrier model were extremely high, which was unreasonable for representing the transportation kinetics. Thus, the single-carrier model was selected to represent the D-xylose transport for induced *C. sorokiniana*. Non-linear regression analysis showed that the maximum D-xylose transport rate was 3.8 nmol min^−1^ mg^−1^ dry cell weight (DCW)with *K*_*m*_ value of 6.8 mM.

**Table 1:** **Kinetic parameters for D-xylose transport in** ***C. sorokiniana***

	**Induced**		**Non-induced**	
**Parameter**	**Model I**	**Model II**	**Model I**	**Model II**
*V*_*max1*_ (nmol min^−1^ mg^−1^ DCW)	3.8	3.0	0.3	0.1
*V*_*max2*_ (nmol min^−1^ mg^−1^ DCW)	-	1.9 × 10^6^	-	9.0 × 10^4^
*K*_*m1*_ (mM)	6.8	4.7	5.5	0.6
*K*_*m2*_ (mM)	-	1.2 × 10^8^	-	2.2 × 10^7^
Variance	3.6 × 10^−2^	3.7 × 10^−2^	1.7 × 10^−3^	4.0 × 10^−4^

Non-induced *C. sorokiniana* also exhibited the capability to take up D-xylose, but with a comparatively low uptake rate. For instance, the uptake rate of 2.0 nmol min^−1^ mg^−1^ DCW was observed at 5 mM D-xylose for the induced algae, while the non-induced cells only showed a rate of 0.1 nmol min^−1^ mg^−1^ DCW. The two-carrier model gave a lower variance than the single-carrier model for the non-induced algal cells, but the *V*_*max2*_ and *K*_*m2*_ values were too high to make the model reasonable. According to the kinetic parameters of the single-carrier model, the maximum D-xylose uptake rate was only 0.3 nmol min^−1^ mg^−1^ DCW, 10 times lower than that of the induced algae, but the *K*_*m*_ value (5.5 mM) was almost at the same level. Thus, these results indicated that the transporters had comparable affinities to D-xylose for the algae regardless of induction, however, the induced cells exhibited a much higher capacity.

### Effect of mono-sugars on D-xylose uptake

The effects of different mono-sugars on D-xylose uptake in the microalga *C. sorokiniana* are shown in Table 2. D-xylose uptake rate was significantly inhibited in the presence of D-glucose, independent of the induction modes, which indicated that D-glucose competed with D-xylose for the transporter and had a much higher affinity. Similarly, D-galactose also functioned as a competitor to the transporter, but it showed much stronger inhibition in the non-induced cells. A different inhibitory manner was observed on D-fructose, which did not inhibit D-xylose uptake in the non-induced algae but slightly suppressed the transport in the induced cells. The pentose sugars (L-arabinose and D-ribose) did not play a significant negative role in D-xylose uptake for *C. sorokiniana*, which might be attributed to the low affinity or even that the transporter lacked the capability to take up these two sugars.

**Table 2:** **Effect of mono-sugars on the rate of D-xylose transport by** ***C. sorokiniana***

	**Induced**		**Non-induced**	
	***V*** **(nmol min** ^**−1**^ **mg** ^**−1**^ **DCW)**	**Inhibition** ^**a**^ **(%)**	***V*** **(nmol min** ^**−1**^ **mg** ^**−1**^ **DCW)**	**Inhibition (%)**
D-glucose	0.044	92.4	0.011	89.4
D-galactose	0.134	76.7	0.003	97.0
D-fructose	0.504	12.5	0.120	0.0
L-arabinose	0.583	0.0	0.140	0.0
D-ribose	0.567	1.5	0.128	0.0

^a^Percentage of inhibition was calculated based on the control without addition of other mono-sugars.

### D-xylose metabolic enzymes

The activities of xylose reductase (XR) and xylitol dehydrogenase (XDH) present in crude cell-free extract of *C. sorokiniana* cultivated under different conditions are shown in Table 3. When the algal cells grew on D-glucose, the enzyme activity was not detectable, independent of induction modes. After incubated with D-xylose for 24 hours, the induced *C. sorokiniana* exhibited XR and XDH activities of 0.021 and 0.026 U mg^−1^ protein respectively, indicating that the expression of XR and XDH was induced by D-xylose. However, the enzyme activity was not detected for the non-induced algae although the cells were capable of taking up D-xylose. The reason might be the enzyme activity was too low to be detected or the intracellular D-xylose was not sufficient to induce the enzyme expression due to the low sugar uptake rate.

**Table 3:** **The activity of XR and XDH present in crude cell-free extract obtained from disrupted cells of** ***C. sorokiniana*** ^**a**^

	**Induced**		**Non-induced**	
	**D-xylose**	**D-glucose**	**D-xylose**	**D-glucose**
XR^b^ (U mg^−1^ protein)	0.021	ND	ND	ND
XDH^c^ (U mg^−1^ protein)	0.026	ND	ND	ND

^a^The non-induced and induced algal cells were washed with sterile distilled water and re-suspended in 8 mg DCW per mL in 50 mL minimal medium supplemented with 5 mM KNO_3_ and 40 mM D-xylose or D-glucose under light for 24 hours.

^b^The activity of XR was measured at pH 6.0 and 40°C with NADPH as a cofactor.

^c^The activity of XDH was measured at pH 8.0 and 40°C with NADP^+^ as a cofactor.

ND, Not detected.

**Figure 2:**
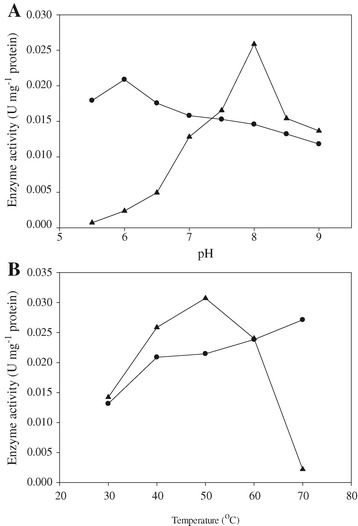
**Effect of pH (A) and temperature (B) on the activity of XR (circle) and XDH (triangle) present in crude cell-free extract obtained from induced** ***C. sorokiniana*** **.** The induced algal cells were washed with sterile distilled water and re-suspended in 8 mg DCW per mL in 50 mL minimal medium supplemented with 5 mM KNO 3 and 40 mM D-xylose under light for 24 hours.

Figure 2 shows the effects of pH and temperature on the activity of XR and XDH, present in crude cell-free extract obtained from the induced *C. sorokiniana* after incubation with D-xylose. The activity of XR increased from 0.018 to 0.021 U mg^−1^ protein with an increase in pH from 5.5 to 6.0, followed by a decrease to 0.012 U mg^−1^ protein at pH 9.0. In the same pH range tested, XDH achieved the highest activity of 0.026 U mg^−1^ protein at pH 8.0. At pH 6.0 (the optimal pH for XR), the activity of XDH decreased 92% from its highest value. Meanwhile, a loss of only 30% was observed on XR activity at pH 8.0 (the optimal pH for XDH). Therefore, an acidic environment was more detrimental to XDH than the negative effect of alkalinity to XR. The optimal temperature for XDH was 50°C and further increasing the temperature to 70°C resulted in an activity loss of 93%. XR was more stable at higher temperature than XDH. The elevated temperature improved the performance of XR and the highest activity of 0.027 U mg^−1^ protein was obtained at 70°C.

### Cofactors for xylose reductase and xylitol dehydrogenase

Table 4 shows the activities of XR and XDH present in crude cell-free extract of the induced *C. sorokiniana* after incubation with D-xylose with different cofactors. Both NADPH and NADH could be used as the cofactor for the reaction from D-xylose to xylitol catalyzed by XR, but the enzyme had a preference for NADPH. The activity of XR with NADPH was about 5-fold higher than that with NADH. As opposed to XR, XDH could catalyze the oxidation of xylitol to D-xylulose only in the presence of the cofactor NADP^+^.

**Table 4:** **The activity of XR and XDH of** ***C. sorokiniana*** **with different cofactors** ^**a**^

	**NADPH**	**NADH**	**NADP** ^**+**^	**NAD** ^**+**^
XR^b^ (U mg^−1^ protein)	0.021	0.004	-	-
XDH^c^ (U mg^−1^ protein)	-	-	0.026	ND

^a^The induced algal cells were washed with sterile distilled water and re-suspended in 8 mg DCW per mL in 50 mL minimal medium supplemented with 5 mM KNO_3_ and 40 mM D-xylose under light for 24 hours.

^b^The activity of XR was measured at pH 6.0 and 40°C.

^c^The activity of XDH was measured at pH 8.0 and 40°C.

ND, Not detected.

### Effect of light on D-xylose utilization

**Figure 3:**
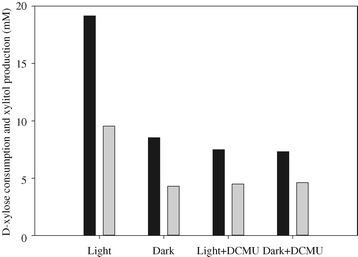
**D-xylose consumption (dark column) and xylitol production (white column) by** ***C. sorokiniana*** **.** The induced algal cells were washed with sterile distilled water and re-suspended in 8 mg DCW per mL in 50 mL minimal medium supplemented with 5 mM KNO 3 and 40 mM D-xylose under different conditions for 24 hours.

Figure 3 shows D-xylose consumption and xylitol production by the induced *C. sorokiniana* under different conditions. The highest D-xylose consumption of 19.1 mM was observed for the cultures with light. *C. sorokiniana* also had the capability to utilize D-xylose in the dark but the consumption was more than 2-fold lower compared with the cultures under light. The stimulation of D-xylose utilization by light was diminished in the presence of 3-(3,4-dichlorophenyl)-1,1-dimethylurea (DCMU), evidenced by a comparable D-xylose consumption with the cultures in the dark. However, DCMU did not play a significant inhibitory role in D-xylose utilization in the dark conditions. Xylitol was formed during the growth in all the cultures. *C. sorokiniana* converted 50 to 60% of the consumed D-xylose to xylitol.

## Discussion

The first step for D-xylose metabolism is the uptake of the sugar through the plasma membrane. A lack of transport system may be one of the major reasons for microalgae without the capability to utilize D-xylose as the sole carbon source. There are two kinetically distinct D-xylose transport systems - the high-affinity system (specific for D-xylose) and the low-affinity system (shared with D-glucose) for native D-xylose-metabolizing microbes. Meanwhile, D-xylose also can be taken up by the D-glucose transporters for non-native D-xylose utilizing species, such as the ethanol-producing yeast *Saccharomyces cerevisiae* [21].

**Figure 4:**
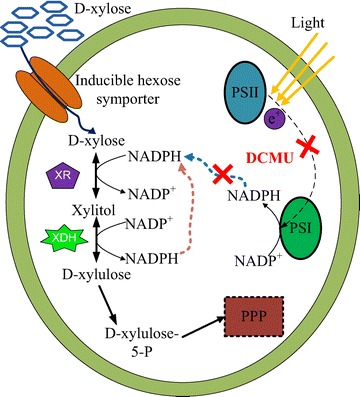
**Proposed D-xylose metabolic pathway in the green microalga** ***C. sorokiniana*** **.** D-xylose is transported across the cell membrane through the inducible hexose symporter. The uptake of D-xylose activates the expression of NADPH-linked XR and NADP + -linked XDH, which converts D-xylose to D-xylulose and enters the PPP pathway after catalyzing to D-xylulose 5-P. NADPH generated during the first stage of photosynthesis from light energy serves as the coenzyme for D-xylose metabolism. DCMU negatively affects the improvement of D-xylose catabolism from the light-dependent reaction by blocking electron flow from photosystem II (PSII) to photosystem I (PSI) and inhibiting NADPH production.

Our results showed that *C. sorokiniana* could take up D-xylose through a single-carrier system. The maximum D-xylose transport rate of 3.8 nmol min^−1^ mg^−1^ DCW with *K*_*m*_ value of 6.8 mM was obtained in D-glucose induced *C. sorokiniana*. It has been reported that *Chlorella* cells possess an inducible active hexose/H^+^ symport system responsible for uptake of D-glucose from the environment [12]. Similar with the D-xylose-metabolizing microbes, the green microalga *C. sorokiniana* might share the inducible hexose symporter for D-xylose uptake (Figure 4). However, D-glucose strongly inhibits D-xylose transportation due to the different affinities for these two sugars [21]. Our results demonstrated that D-xylose uptake was suppressed in the presence of D-glucose, D-galactose and D-fructose, but the degrees of inhibition were different due to the different affinities to these hexoses. *C. sorokiniana* might not be able to transport the pentose sugars (L-arabinose and D-ribose) by using the hexose symporter, because no significant inhibitory effect on D-xylose assimilation was observed for these sugars.

Interestingly, the non-induced *C. sorokiniana* were also able to take up D-xylose and had similar affinity (*K*_*m*_) to D-xylose compared with the induced cells. Thus, the non-induced cells might still utilize the hexose symporter for D-xylose assimilation, which was also evidenced by the severe inhibition of D-xylose uptake in the presence of D-glucose and D-galactose. However, the capacity (*V*_*max*_) was much lower than that of the induced algae, indicating a limited quantity of transporters present in the non-induced cells. Tanner [12] suggested that a small amount of transport protein was constitutively present in the algal membrane. Haass and Tanner [13] reported that non-induced *Chlorella* strains had the sugar uptake capability and the uptake rate increased from 2 to 412-fold after induction by D-glucose (D-glucose induced cells). This was in accordance with our results of D-xylose uptake for the non-induced cells, and the data also demonstrated that D-glucose induced *C. sorokiniana* exhibited a remarkably increased D-xylose uptake rate.

After D-xylose is transported into the cells, it will be reduced to xylitol by XR and then converted to D-xylulose by XDH in most eukaryotic microorganisms. Subsequently, D-xylulose is converted to D-xylulose 5-phosphate (D-xylulose 5-P) by xylulokinase before entering the PPP pathway [22]. In this study, NAD(P)H-linked XR and NADP^+^-linked XDH activities were detected in crude cell-free extract obtained from the induced *C. sorokiniana* after incubation with D-xylose. In fact, XDH from reported wild-type microorganisms exclusively uses NAD^+^ as the cofactor, while XR prefers NADPH [22–24]. The imbalance of redox cofactors will occur due to the different coenzyme usage, resulting in high amounts of xylitol accumulation. This study discovered a unique XDH from the microalga *C. sorokiniana*, which oxidized xylitol to form xylulose using only NADP^+^ as a cosubstrate.

Our results still showed that a notable quantity of xylitol was produced during the growth of *C. sorokiniana* on D-xylose. The imbalance of cofactors is not the reason for this because the XDH of *C. sorokiniana* utilizes NADP^+^ as the cofactor, while the XR prefers NADPH. The formation of xylitol is not solely a consequence of coenzyme imbalance and some metabolic factors may also cause its production, such as the activity ratios of XR and XDH. It has been reported that a high ratio of 10:1 of XDH and XR activities were essential to improve the conversion of D-xylose to D-xylulose [25]. However, our data showed that the activity ratio of XDH and XR was only 1.8:1 at pH 8.0 (optimal pH of XDH). Actually, *Chlorella* tends to maintain an intracellular pH relative constant around 7.0 [26,27]. Under this condition, the activity of XR was even higher than XDH (Figure 2A) and the equilibrium of the reaction favored xylitol formation.

Unlike yeast and fungi, microalgae are photosynthetic organisms and have the capability to capture light energy through photochemical reactions. During the first stage of photosynthesis, light energy is converted into chemical energy such as NADPH, which can potentially serve as the coenzyme for D-xylose metabolism (Figure 4). Our results showed that the D-xylose consumption was 2-fold higher under light compared with the cultures in the dark. However, in the presence of DCMU, an herbicide specifically blocking electron flow from photosystem II and inhibiting NADPH production, the consumption of D-xylose was almost identical with that of the cultures in the dark. This result also reflected that the improvement of D-xylose utilization by light might be attributed to the extra chemical energy from the light-dependent reaction.

Although *C. sorokiniana* could take up D-xylose through the inducible hexose symporter and express XR and XDH for D-xylose catabolism, the growth efficiency was very low since the cell number and DCW did not obviously increase. There are several possible reasons for this phenomenon. The synthesis of the hexose symporter could be induced by D-glucose, D-galactose and D-fructose, but the induction was not achievable by pentoses [12]. Although D-glucose induced algae can take up D-xylose efficiently, the reproduced algal cells may not possess such a transport system (or only a very few amount of constitutive transporters), which subsequently hinders the expression of XR and XDH and eventually results in the death of the reproduced cells. Hahn-Hägerdal *et al*. [22] declared that the inability of *S. cerevisiae* to utilize D-xylose was due to the low expression of XR and XDH although the genes encoding these enzymes were present in its genome. Additionally, the low activity ratio of XDH and XR limits D-xylose toward the central metabolism. Our data showed that more than half of the D-xylose carbon was secreted out in the form of xylitol, which would lead to inefficient ATP generation [28]. Microalgae require a significant amount of ATP just for maintenance [6]. As a result, the energy and carbon are inadequate to support the cell reproduction and biomass accumulation.

As natural photosynthetic microorganisms, most microalgae can only grow photoautotrophically, but some of them can grow heterotrophically and/or mixotrophically using organic substrates, which shows great potential in producing biofuels and valuable chemicals due to the rapid growth [2]. Generally, D-glucose is the most preferable organic carbon for these species. D-xylose is hardly utilized by many microbes as the sole carbon source due to a lack of efficient uptake system and/or the catalytic enzymes. Many reports have stated that microalgae could not grow on D-xylose [16–18]. However, our present work shows different results. The microalga *C. sorokiniana* could not only assimilate D-xylose from the environment but also had the capability to catalyze it. This is the first time that D-xylose metabolic enzymes in microalgae have been identified. It is meaningful for future genetic engineering since the introduction of an entire D-xylose metabolic pathway from other microbes into the microalga *C. sorokiniana* is not necessary. Additionally, *C. sorokiniana* expresses a unique XDH using NADP^+^ as the cofactor, which has not been reported in any wild-type microorganisms [22–24]. The utilization of NADP^+^ as cofactor is quite significant since XR prefers NADPH, resulting in balanced redox cofactors. This unique XDH has potential to be applied in the genetic modification of some typical strains, such as the ethanol producing yeast *S. cerevisiae*, to overcome the imbalance of redox cofactors during fermentation of D-xylose.

In conclusion, the presented results demonstrated that the oleaginous microalga *C. sorokiniana* (UTEX 1602) had the capability to utilize D-xylose. The analysis of sugar uptake kinetics suggested that the inducible hexose symporter might be responsible for the transport of D-xylose across the cell membrane. The enzymatic activities of NAD(P)H-linked XR and NADP^+^-linked XDH were detected in *C. sorokiniana* after the assimilation of D-xylose. Culturing *C. sorokiniana* under light could improve D-xylose utilization due to extra NADPH from the light-dependent reaction of photosynthesis. This study provided a new insight into D-xylose metabolism in microalgae. Future work will continue to identify the key enzymes and related genes of D-xylose metabolic pathway in *C. sorokiniana*, and genetic engineering will be adopted to improve D-xylose utilization, for example by introducing a D-xylose specific transporter and overexpressing XDH and XR in a reasonable ratio.

## Materials and methods

### Organism, media and cultivation

The green microalga *C. sorokiniana* (UTEX 1602) was obtained from the Culture Collection of Algae at the University of Texas (Austin, Texas, United States). The minimal medium used in all the cultivations was according to our previous description [20]. Flask cultures were conducted in 250 mL Erlenmeyer flasks. Phototrophic cultures contained 200 mL minimal medium supplemented with 10 mM KNO_3_ and were bubbled with air (1.0% CO_2_) at a rate of 100 mL min^−1^. The vessels were illuminated with continuously fluorescence light at a photon flux density of 100 μmol m^−2^ s^−1^. Algal cells were harvested at the exponential phase and used as non-induced cells in following experiments. Heterotrophic cultures containing 50 mL minimal medium were supplemented with 20 mM KNO_3_ and 100 mM D-glucose, and incubated at a rotary rate of 200 rpm in dark. Algal cells were harvested at the exponential phase and used as D-glucose induced cells in following experiments. All the media used in this study were sterilized by passing through a 0.22 μm membrane (Millipore, Massachusetts, United States).

### ^14^C-labeled D-xylose uptake assay

The method for ^14^C-labeled D-xylose uptake assay was carried out according to the description by Du *et al*. [29]. ^14^C-labeled D-xylose was obtained from American Radiolabeled Chemicals (St. Louis, Missouri, United States). Non-induced and induced algal cells were washed with ice-cold water three times. The cells were then re-suspended in about 60 mg DCW per mL in potassium phosphate buffer (50 mM, pH 7.0). A total of 1 mL cell suspension was dried in a pre-weighed aluminum dish at 105°C for 3 hours to determine the DCW. The prepared cell suspension was kept on ice before use. D-xylose uptake kinetics was initiated by mixing 160 μL cell suspension (5 minutes pre-incubation at 37°C) with 40 μL labeled D-xylose of various concentrations. Sugar inhibition studies were performed with 20 μL labeled D-xylose (5 mM) and 20 μL unlabeled sugar (5 mM). The reaction was stopped by adding 10 mL ice-cold water after 30 seconds. The zero time point was handled by adding cell suspension and ice-cold water simultaneously. The mixture was filtered through a Whatman GF/C filter (Whatman, New Jersey, United States) presoaked in 40% D-xylose and washed twice with 10 mL ice-cold water. Samples were counted with a Beckman LS6500 scintillation counter (Beckman Coulter, California, United States) in the scintillation cocktail (National Diagnostics, Georgia, United States). Three independent assays were used to determine kinetic values. The uptake rate was recorded as nanomoles (nmol) of labeled D-xylose min^−1^ mg^−1^ of DCW.

### Kinetic analysis

The sugar uptake data were analyzed with non-linear regression using SigmaPlot 12.0 (Systat Software, California, United States). The analysis was performed with two models [30]. Model I, Michaelis-Menten function for a single carrier:

1 $$ V={V}_{max1}\bullet \left[S\right]/\left({K}_{m 1}+\left[S\right]\right) $$

Model II, Michaelis-Menten function for two carriers:

2 $$ V={V}_{max1}\bullet \left[S\right]/\left({K}_{m 1}+\left[S\right]\right)+{V}_{max2}\bullet \left[S\right]/\left({K}_{m 2}+\left[S\right]\right) $$

where *K*_*m1*_ and *V*_*max1*_ indicate *K*_*m*_ value and maximum D-xylose transport rate for one transporter, while *K*_*m2*_ and *V*_*max2*_ are the corresponding parameters for another transporter.

### D-xylose utilization under light and dark conditions

The non-induced and induced algal cells were washed with sterile distilled water and re-suspended in 8 mg DCW per mL in 50 mL minimal medium supplemented with 5 mM KNO_3_ and 40 mM D-xylose. The cultures were performed in 250 mL Erlenmeyer flasks and shaken with or without light (continuous fluorescence light at a photon flux density of 100 μmol m^−2^ s^−1^) for 24 hours at 37°C. To evaluate the effect of photosynthesis on D-xylose utilization, DCMU was added to the cultures at the concentration of 10 μM.

### Enzyme assay

After incubation with 40 mM D-xylose or D-glucose for 24 hours, the non-induced and induced algal cells were harvested and washed with ice-cold water three times. The cell extracts for enzyme assay were prepared by disrupting the cells with glass beads in 50 mM potassium phosphate buffer (pH 7.0) containing 0.5 mM DTT and 1 mM phenylmethylsulfonyl fluoride. The method for enzyme assay was according to the description by Freer *et al*. [31]. XR activity was assayed in reaction mixtures (1.0 mL) containing 50 mM potassium phosphate buffer (pH 5.5 to 7.0) or Tris buffer (pH 7.5 to 9.0), 50 mM D-xylose, 0.34 mM NAD(P)H and 0.1 mL enzyme preparation. XDH was assayed in a similar manner, except 50 mM xylitol and 2 mM NAD(P)^+^ was used in the reaction mixture. Temperature ranged from 30°C to 70°C. The oxidation or formation of NAD(P)H was recorded at 340 nm using a UV-vis spectrophotometer (UV-2550, Shimadzu, Kyoto, Japan). Background enzyme activities without addition of cofactor were also measured. One unit of enzyme activity was defined as one micromole (μmol) of cofactor converted per minute. Specific enzyme activity was expressed as U mg^−1^ protein, based on the determination of protein concentration according to the procedure described by Bradford [32] using bovine serum albumin as the standard.

### Other analysis

D-glucose, D-xylose and xylitol were analyzed using a Dionex ICS-3000 ion chromatography (Dionex Corp., California, United States), which was equipped with a CarboPac TM PA 20 (4 × 50 mm) analytical column, and CarboPac TM PA 20 (3 × 30 mm) guard column [33]. The filtered samples were injected and eluted isocratically with 0.01 M NaOH. Analytes were detected and quantified based on standard curves. The cell number was determined by a hemacytometer. All the flasks cultures were repeated in triplicate. The experimental data were statistically analyzed with one way analysis of variance (ANOVA) using SAS 9.2 (SAS Institute, North Carolina, United States). All values were presented as the average of independent measurements with significance declared at *P* <0.05.

## Abbreviations

DCMU: 3-(3,4-dichlorophenyl)-1,1-dimethylurea
DCW: Dry cell weight
D-xylulose 5-P: D-xylulose 5-phosphate
EMP: Embden-Meyerhof Pathway
PPP: Pentose phosphate pathway
PSI: Photosystem I
PSII: Photosystem II
XDH: Xylitol dehydrogenase
XR: Xylose reductase
